# Rabies elimination in the WHO African Region

**DOI:** 10.2471/BLT.25.293867

**Published:** 2025-09-01

**Authors:** Jakob Zinsstag, Yewande Alimi, Thomas C Mettenleiter, Hiver Boussini, Huyam Salih

**Affiliations:** a Swiss Tropical and Public Health Institute, Kreuzstrasse 2, 4123 Allschwil, Switzerland.; b Africa Centres for Disease Control and Prevention, Addis Ababa, Ethiopia.; c Friedrich-Loeffler-Institut, Federal Research Institute for Animal Health, Greifswald-Insel Riems, Germany.; d InterAfrican Bureau for Animal Resources, Nairobi, Kenya.

This year, Gavi, the Vaccine Alliance will roll out a rabies vaccine campaign in selected first-adopter countries, after a two-year delay caused by the coronavirus disease 2019 pandemic.^1^ More than 100 years after Louis Pasteur developed post-exposure prophylaxis for rabies, after the roll-out phase, all Gavi-eligible countries will have free access to post-exposure prophylaxis rabies vaccines to meet their needs. In many parts of Africa, rabies-exposed patients still lack access to live-saving post-exposure prophylaxis due to limited availability, high costs of vaccines and the catastrophic household expense they generate. Gavi will thus contribute to filling a gap that still causes the death of over 21 000 people, mostly children, in the African continent every year.^2^ Gavi’s engagement could considerably contribute to addressing access inequities and to reaching the goal of zero human rabies by 2030. The goal was set in 2015 by the World Health Organization (WHO), World Organisation for Animal Health, Food and Agriculture Organization (FAO), and Global Alliance for Rabies Control, working together as United Against Rabies. However, to achieve this objective, eligible countries and Gavi need to make coordinated and quick efforts. 

While improved post-exposure prophylaxis is critical, it cannot stop rabies transmission by itself. Rabies in humans originates mainly from dogs, the most important rabies reservoir, and post-exposure prophylaxis for humans does not affect transmission among dogs. In endemic settings, if rabies continues to be transmitted, the cumulative cost of post-exposure prophylaxis, estimated at about 40 United States dollars (US$) per full course, for the next 30 years is projected at US$ 500–600 million in the African continent alone.^3^ A strategic mass dog vaccination coordinated within and between countries alongside post-exposure prophylaxis could eliminate dog rabies in Africa, generating total welfare gains of US$ 9.5 billion between 2024 and 2054.^4^ A coordinated mass dog vaccination offers a return on investment of 14 to 1. For example, Niger could see potential welfare gains of up to 2.5%, (US$ 488 million) of its gross domestic product.^4^ Coordinated rabies control between African countries could lead to more socially and ecologically equitable outcomes by reducing the number of lost human lives to almost zero and possibly eliminating the disease.

Most dogs in Africa are free-roaming but owned and therefore accessible for vaccination. In addition to parenteral vaccines, oral vaccination of dogs can substantially increase vaccination coverage and accelerate control. Safe, effective and affordable rabies vaccines for dogs, developed in line with World Organization of Animal Health standards, are available. Based on research evidence^5^ and following the example of the global elimination of rinderpest in 2011, the elimination of dog rabies in Africa appears to be feasible through coordination among African countries. The InterAfrican Bureau for Animal Resources, Pan African Veterinary Vaccine Centre and Regional Economic Communities are currently preparing a roadmap for a coordinated Pan-African Rabies Elimination Campaign.^6^

The engagement of the animal health sector, led by the InterAfrican Bureau for Animal Resources, is now complemented by the Africa Centres for Disease Control and Prevention (CDC) leadership of the African Union’s One Health coordination group on zoonotic diseases.^7^ These agencies have collectively developed the *One Health zoonotic disease prevention and control strategy 2025–2029*,^8^ through which the African Union’s technical agencies are strengthening coordination mechanisms across Member States for effective rabies elimination. This initiative aligns with the aspirations of the African Union’s *Agenda 2063: the Africa we want* and with the *One Health joint plan of action* – action track 3 of the Quadripartite organizations (WHO, FAO, World Organisation for Animal Health and United Nations Environment Programme).^9^ All tools are therefore available to reach the goal of zero human rabies by 2030, for the benefit of human and animal health, if relevant stakeholders ensure coordination, cooperation, communication and capacity-building. 

Leveraging the African Union’s convening power, the Africa CDC and InterAfrican Bureau for Animal Resources are mobilizing political support^6^ for One Health coordination at subnational, national and regional levels to improve African countries’ ability to eliminate rabies by 2030.^10^
